# The stoneflies (Plecoptera) of Arkansas: a checklist compiled from museum specimen data

**DOI:** 10.3897/BDJ.13.e145637

**Published:** 2025-02-26

**Authors:** Lily Veronica Hart, Ralph Edward DeWalt, Phillip N. Hogan, Scott A. Grubbs, David K. Burton

**Affiliations:** 1 Department of Entomology, University of Illinois, Urbana-Champaign, United States of America Department of Entomology, University of Illinois Urbana-Champaign United States of America; 2 Illinois Natural History Survey, University of Illinois, Urbana-Champaign, United States of America Illinois Natural History Survey, University of Illinois Urbana-Champaign United States of America; 3 Department of Biology and Center for Biodiversity Studies, Western Kentucky University, Bowling Green, United States of America Department of Biology and Center for Biodiversity Studies, Western Kentucky University Bowling Green United States of America; 4 Canadian National Collection of Insects, Ottawa, Canada Canadian National Collection of Insects Ottawa Canada

**Keywords:** aquatic insects, biodiversity, Species of Greatest Conservation Need, State Wildlife Action Plan, TaxonWorks

## Abstract

**Background:**

Stoneflies are well known as indicators of water quality. Their presence in running waters, glacial meltwaters, and large oligotrophic lakes is rapidly declining the world over. In the USA, states partner with the U.S. Fish and Wildlife Service to protect habitat and wildlife through the development of State Wildlife Action Plans (SWAPs). Plants and wildlife species often enter these SWAPs as Species in Greatest Conservation Need (SGCN). Arkansas currently lists nine stonefly species as SGCNs and has funded research on them through SWAP grants. However, these nine species were initially chosen based on the small amount of data from a few papers. A more comprehensive assessment using museum specimen data is necessary to assess completeness of sampling, the relative rarity and endemicity of species, temporal changes in distribution, and the conservation status of species in Arkansas. Herein, we publish a data paper and preliminary dataset comprised of specimen data primarily from the Illinois Natural History Survey Insect Collection, Canadian National Collection, Western Kentucky University, P. N. Hogan Personal Collection, and from existing literature sources. These data are made publicly available by the Global Biodiversity Information Facility (GBIF) to allow for comprehensive assessment of the Arkansas Plecoptera assemblage. More recent occurrence data are needed to accurately assess imperilment of Arkansas stonefly species; these data will be provided through targeted collecting, collaboration with others in Arkansas, and through investigation of additional museum collections.

**New information:**

This dataset includes > 3,500 specimen records (ethanol vials or pins with or without catalog numbers) and accounts for 84 stonefly species in Arkansas, six more species than indicated in published records. Perlidae contributed 29 of these species followed distantly by Perlodidae (15), Capniidae (14), Taeniopterygidae (9), Leuctridae (7), Chloroperlidae (5), Nemouridae (4), and Pteronarcyidae (1). A species accumulation curve predicts that sampling of species is nearly complete with a Chao1 estimate of 88.0 ± 3.7 species. Our data demonstrate that 25 species are known from ≤ five records, suggesting that many more than the nine recognized stonefly SGCNs in Arkansas may meet standards for inclusion.

## Introduction

U.S. Fish & Wildlife Service (USFWS) funding has helped individual states to develop State Wildlife Action Plans (SWAPs). SWAPs discuss plans for wildlife conservation and enlist partners at local, state, federal and non-governmental organizations (NGOs) to coordinate efforts. The Arkansas Wildlife Action Plan (AWAP) is updated on a 10-year cycle, with the next update due in 2025 (Fowler and Anderson 2015). Research and management of species and habitats are funded through State Wildlife Grants from the Arkansas Fish & Game Commission. These grants aim to identify, protect, and manage Species of Greatest Conservation Need (SGCN). Currently, the state of Arkansas lists nine stonefly (order Plecoptera) species as SGCN. However, to update this list, the state needs a complete dataset, since current data are based upon historical literature (e.g. Poulton and Stewart (1987), Poulton and Stewart (1991)) summarized from thousands of specimens scattered among several institutional and private collections. Access to such a dataset will aid research scientists, regional taxonomists, and agency managers to assess completeness of sampling, conservation status, and temporal shifts in the distribution of Arkansas stonefly species. As of yet, no such dataset exists for the state of Arkansas.

This project is an attempt to create a comprehensive stonefly specimen dataset for the state of Arkansas. We have created a preliminary species checklist, based on > 3,500 records from the Illinois Natural History Survey Insect Collection (INHS), the Canadian National Collection of Insects, Arachnids, and Nematodes (CNCI), Western Kentucky University Insect Collection (WKU), and several other institutional, personal, and literature records (Table 1), similar to other biodiversity studies using specimen-based data (Guevara et al. 2024). Over the next two years, we will add more specimen records through targeted sampling, compiling additional records from collections not yet accessed (e.g. Arkansas State University, Brigham Young University, Colorado State University, Smithsonian National Museum of Natural History), and through collaboration with colleagues at the University of Arkansas, Fayetteville. This data paper illustrates our intention to continue faunistics work within the state. The initial and most utilitarian product of this manuscript is the dataset that may be cited by others and updated as our work progresses.

### History of Plecopterology in Arkansas

Some of the earliest records for Arkansas stoneflies appear in Needham and Claassen (1925), who tallied two genera and the species *Perlinellaephyre* (Newman, 1839) and *Acroneuriaarida* (Hagen, 1861), the latter most certainly referable to *A.frisoni* Stark & Brown, 1991. Frison (1942) presented paratype data for the newly-described *Taeniopteryxlita* Frison, 1942 and the first records for *Nemocapniacarolina* Banks, 1938, *Haploperlabrevis* (Banks, 1895) and *Alloperlacaudata* Frison, 1934. Ricker (1952) erected many new subgenera in multiple families for the Nearctic fauna, most elevated to generic status by Illies (1966). Ricker (1952) also presented paratype records for *Amphinemuradelosa* (Ricker, 1952), stated without citation the presence of *Prostoiacompleta* (Walker, 1852), *Hydroperlacrosbyi* (Needham & Claassen, 1925), *H.fugitans* (Needham & Claassen, 1925), *Clioperlaclio* (Newman, 1839), and described, from Arkansas, specimens of the winter stonefly *Allocapniasandersoni* Ricker, 1952.

Work conducted in the 1960s and 1970s contributed 11 *Allocapnia* Claassen, 1928 species to the Arkansas record (Ross 1964, Ross and Ricker 1971). The addition of *A.oribata* by Poulton and Stewart (1987) was the last to be added to the *Allocapnia* list. Ricker and Ross (1968), in their revision of *Taeniopteryx* Pictet, 1841, described new species with holotypes and/or paratypes or provided additional specimen records of existing Arkansas species: *T.burksi* n.sp., *T.lita*, *T.maura* (Pictet, 1841), *T.metequi* n.sp., and *T.parvula* Banks, 1918. This prolific pair also produced a revision of *Zealeuctra* Ricker, 1952 and contributed records for three new and one existing species: *Z.claasseni* (Frison, 1929), *Z.narfi* n.sp., *Z.wachita* n.sp., and *Z.warreni* n.sp. (Ricker and Ross 1969). Their work continued with a world review of the Brachypterainae (their Brachypterinae) that described two new species of *Strophopteryx* Frison, 1929 from Arkansas: *S.arkansae* and *S.ostra* (a syn. of *S.cucullata* Frison, 1934) (Ricker and Ross 1975).

**Figure 1.**
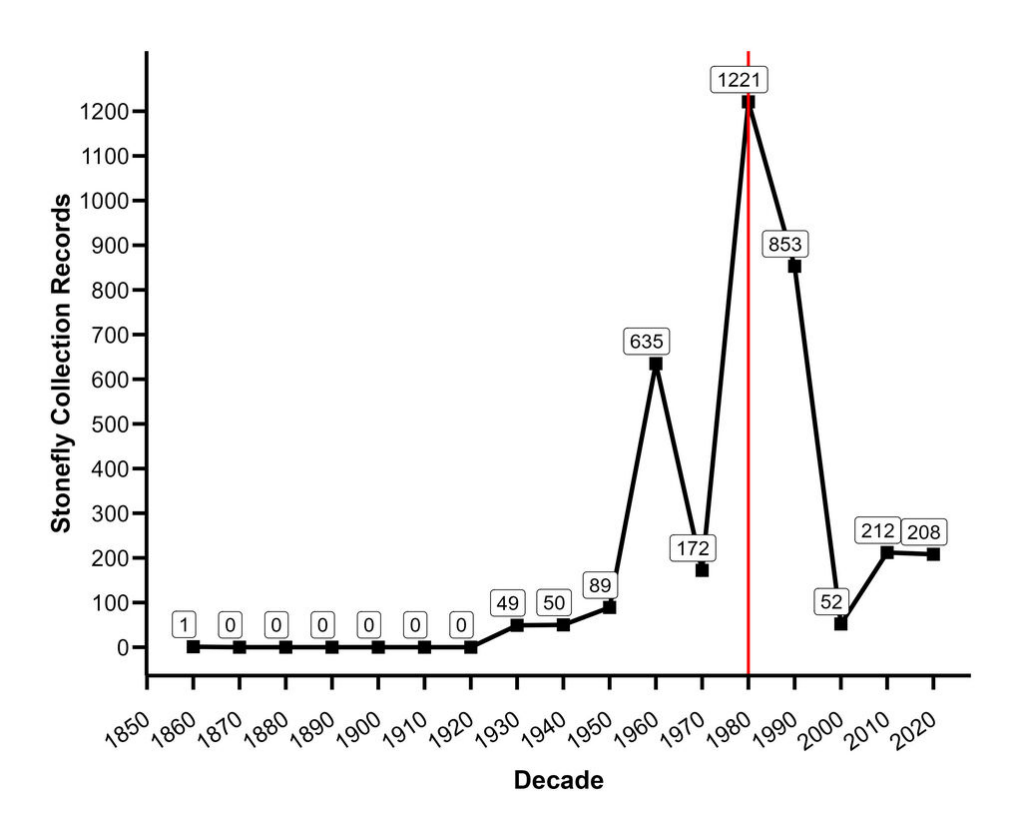
Collections of stoneflies in Arkansas per decade. The vertical red line represents the decade 1980s.

Collecting efforts in the decades of the 1980s and early 1990s ramped up dramatically (Fig. 1). The efforts of Poulton and Stewart (1987) and Poulton and Stewart (1991) resulted in the description of six new species (*Allocapniaoribata* Poulton & Stewart, 1987; *Leuctrapaleo* Poulton & Stewart, 1991; *Alloperlacaddo* Poulton & Stewart, 1987; *Isoperlaszczytkoi* Poulton & Stewart, 1987; *Acroneuriaozarkensis* Poulton & Stewart, 1991; and *Perlestafusca* Poulton & Stewart, 1991). Poulton and Stewart (1991) also provided illustrated keys to adults and larvae, and summarized the distribution of 88 stonefly species from the Ozark-Ouachita region of Arkansas, Illinois, Missouri, and Oklahoma. Unfortunately, they did not explicitly list species occurrences by state in their table of Ozark-Ouachita Plecoptera. Our examination of watershed records found 78 Arkansas species.

Harp and Robison (2006) documented seven endemic stoneflies to occur in the Strawberry River system (part of the Arkansas Natural and Scenic Rivers System), out of the 25 regional endemics listed in Poulton and Stewart (1991). *Attaneuriaruralis* (Hagen, 1861) is rarely collected in Arkansas, but Harp and Robison (2006) included it in their list based on larval specimens taken from smaller drainages of the Strawberry River. Given that *A.ruralis* is a large river species (Poulton and Stewart 1991), these records likely represent misidentifications of disjunct *Paragnetina media* (Walker, 1852).

Grubbs and DeWalt (2012) described *Perlestaephelida*, listing Arkansas material, and removed *P.shubuta* Stark, 1989 from the Arkansas list. Robison and McAllister (2018) published an updated checklist that included 77 species. However, they omitted the regional endemic species *Strophopteryxcucullata* Frison, 1934 from the list (Frison 1934, Poulton and Stewart 1991). Recently, *Perlestasublobata* South & DeWalt, 2019 was described from Arkansas and Oklahoma (South et al. 2019). Annaratone et al. (2023) published a study using random forest models to predict habitat and distribution of an endemic winter stonefly, *Allocapniamohri* Ross & Ricker, 1964. Most recently, Hogan et al. (2024) elucidated the distribution of *A.oribata* and provided distribution maps for all *Allocapnia* endemic to the Interior Highlands and South Central Plains Ecoregion.

There have been several nomenclatural changes since the publications of Poulton and Stewart (1991) and Robison and McAllister (2018). Grubbs et al. (2023) confirmed the long-held suspicion that *L.paleo* Poulton & Stewart, 1991 was a junior synonym of *L.szczytkoi* Stark & Stewart, 1981, thus removing *L.paleo* from the Arkansas SGCN list and replacing it with the now more broadly distributed *L.szczytkoi*, known from the South Central Plains Ecoregion of Arkansas, Louisiana and Texas (Table 2).

*Acroneuriamela* Frison, 1942 is now considered a junior synonym of *A.evoluta* Klapálek, 1909, a large river species (Stark and Brown 1991). Poulton and Stewart (1991) used the name *A.evoluta*, but Stark and Brown (1991) demonstrated that most specimens identified as *A.evoluta* by Nearctic workers prior to 1991 were actually an undescribed species that they described as *A.frisoni* Stark & Brown, 1991, a small stream to medium river species. *Amphinemuranigritta* (Provancher, 1876) reported in Poulton and Stewart (1991) and Robison and McAllister (2018) is now known as *A.texana* Baumann, 1996 (Baumann 1996). This finding presumably restricts *A.nigritta* further east, with the exception of its presence in Missouri (Baumann 1996, DeWalt et al. 2024). *Prostoiacompleta* (Walker, 1852), once listed from west of the Mississippi River in Arkansas, Missouri, and Oklahoma, is now referable to *P.ozarkensis* Baumann & Grubbs, 2014 (Grubbs et al. 2014). A distributional change has bearing on the Arkansas endemic and SGCN *Z.wachita* Ricker & Ross, 1969. Grubbs et al. (2013) reported several collections of this species from LeFlore Co., Oklahoma, demonstrating that it is an Ouachita Mountains endemic, but not endemic to Arkansas alone.

Our dataset and species checklist reflect these updates. To date, Poulton and Stewart (1991) remains the most thorough resource for stonefly species level identification, distribution, and life history information within the region. This information is summarized in DeWalt et al. (2024).

### Dataset Description

Arkansas Plecoptera V1 (Suppl. material 1) consists of specimen data from 13 collections and several literature sources (Ross and Ricker 1971, Poulton and Stewart 1987, Poulton and Stewart 1991, Stark 2004, Grubbs et al. 2014, Hogan et al. 2024) (Table 1).

The dataset contains 3,561 records (specimens of one species in vials or on pins with or without a catalog number) of Arkansas stoneflies. Many of these come from donations of stonefly specimens in 2013 from Kenneth W. Stewart (University of North Texas, Denton, deceased), Barry C. Poulton (United States Geological Survey, Columbia, Missouri, retired), and Stanley W. Szczytko (University of Wisconsin, Stevens Point, deceased). Stewart’s collection, resulting from his 50-year career studying stoneflies, was donated to both the INHS (eastern Nearctic material) and Brigham Young University (BYU, western Nearctic material). The Szczytko collection emphasizes the diverse *Isoperla* Banks, 1906 (Perlodidae) (Szczytko and Kondratieff 2015). In 2013, Poulton donated approximately 250 vials. The remainder of Poulton's collection was acquired in 2022 and contained approximately 1,300 vials collected in the 1980s and 1990s, largely as a product of his doctoral work with Stewart and specimens donated by Henry W. Robison (Southern Arkansas University, Magnolia, retired). Without these donations, this dataset would be incomplete. Much of the material represented in this dataset has been previously digitized, imaged, and georeferenced through a grant funded by the U.S. National Science Foundation to help incorporate several large donations of wet collections into the INHS Insect Collection (DeWalt et al. 2018).

### Data methods

The majority of the data compiled for this project comes from museum specimen data at INHS, with the specimen determinations based largely on adult males. Species names were reviewed and validated using Plecoptera Species File (DeWalt et al. 2024). Many specimens were re-examined to ensure that identifications were correct. Particular examples include specimens initially labeled as *Agnetinaannulipes* (Hagen, 1861), *Amphinemuranigritta* (Provancher, 1876), *Hydroperlafugitans* (Needham & Claassen, 1925), *Prostoiacompleta* (Walker, 1852), *Perlestaplacida* (Hagen, 1861), *Neoperlaclymene* (Newman, 1839), and *N.stewarti* Stark & Baumann, 1978. If the names do not appear in Table 2, then the names were updated in the dataset and analyses. Additionally, some specimens in the dataset include an identification qualifier (cf., nr.) due to taxonomic uncertainty and were not included in the species count or data analyses.

The INHS uses the TaxonWorks™ bioinformatics management system to digitize insect collections. TaxonWorks™ is a home-grown, web-based workbench developed for biodiversity scientists and taxonomists (TaxonWorks Community 2022). This workbench is formulated to enhance the storage of specimen data in a relational database capable of handling images, georeferencing locality data, and coordinating literature sources. Each specimen is assigned a unique identifier and is searchable in the database using many filters. TaxonWorks™ can import and export datasets in the standardized Darwin Core Archive (DwC-A) format.

**Figure 2.**
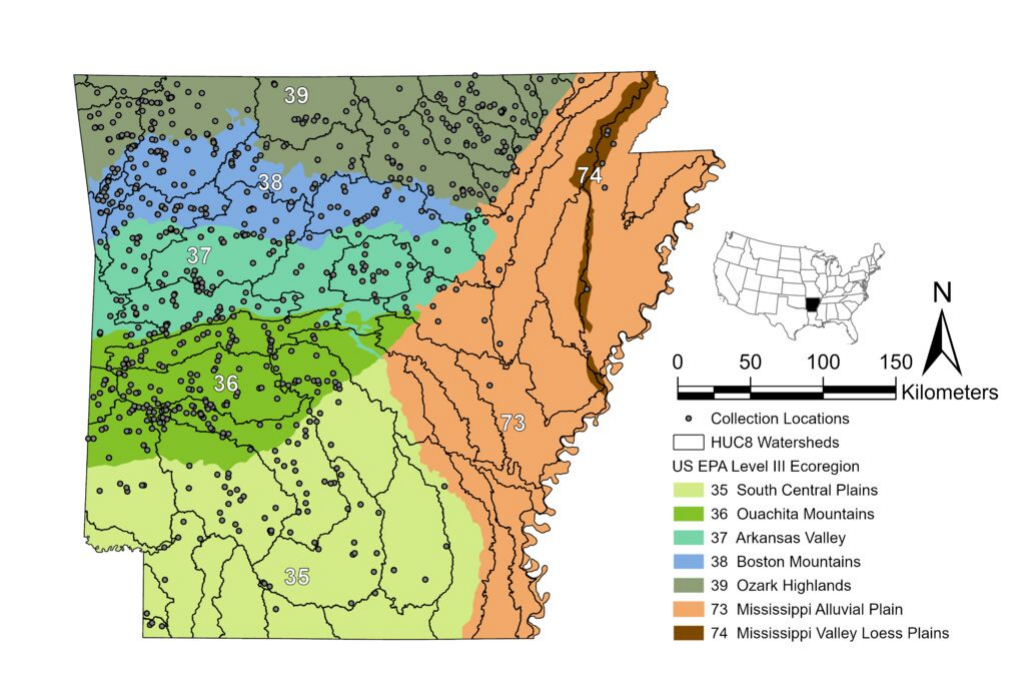
Stonefly collection locations within Arkansas with USGS HUC8 watersheds and US EPA Level III Ecoregions overlaid. Data are projected in WGS84 UTM Zone 15N.

For this project, we filtered all of the Plecoptera specimens in TaxonWorks™ from the spatial geographic extent of the state of Arkansas. This returned > 3,100 records. Next, we checked for completeness to the best of our ability and integrated the various museum specimen records and literature records, for a total of 3,561 records. Data were cleaned using Google Sheets, Microsoft Excel, and OpenRefine (https://openrefine.org/)(Delpeuch et al. 2024), confirming that all data fit the DwC-A standard format (Wieczorek et al. 2012). Data were validated and mapped to this format before being published to GBIF. The completeness of species discovery was analyzed by constructing a matrix of species presence-absence and unique localities (Newman et al. 2021). The intersectional cells were filled with a 1, indicating species presence, or a 0, indicating species absence at that locality. This matrix was imported to RStudio (Posit Team 2024), and the RStudio packages "vegan" (Oksanen et al. 2024) and "ggplot2" (Wickham 2016) were employed. The function *specpool* provided an estimate of species richness, while functions *specaccum* and *ggplot* generated the species accumulation curve. To produce our map (Fig. 2), the data were plotted using ArcGIS (ESRI 2024).

## Sampling methods

### Sampling description

Throughout the course of stonefly research in Arkansas, structured or opportunistic sampling regimens have been utilized. Often, the collecting techniques and effort applied during sampling were not reported. Collection labels are the greatest source of collecting event data, but often lack data on the collecting method utilized. As a result, this dataset contains presence-only records. Common methods employed to collect adult stoneflies include the use of beating sheets and sweep nets along riparian vegetation, handpicking with forceps of adult stoneflies from riparian rocks, vegetation, or bridges, and UV light traps for nocturnal adults. A variety of methods are typically employed to collect larvae including D-frame nets, Surber samplers, or kick nets. Often, nymphal stoneflies were reared to adulthood within a Frigid Units Living Stream unit under simulated light and temperature regimes. When known, the methods used to collect specimens are included in this dataset.

## Geographic coverage

### Description

Low elevation mountains, river valleys, forests, and prairies make Arkansas a biodiversity hotspot in the South Central United States. Arkansas' elevation ranges from 17 m Above Sea Level (m ASL) at its lowest point near the southeastern border of the state, to 839 m ASL at its highest peak at Mount Magazine (United States Geological Survey 2024). There are seven United States Environmental Protection Agency Level III Ecoregions in the state: South Central Plains (35), Ouachita Mountains (36), Arkansas Valley (37), Boston Mountains (38), Ozark Highlands (39), Mississippi Alluvial Plains (73), and Mississippi Valley Loess Plains (74) (USEPA 2024) (Fig. 2). Ecoregions categorize geographic areas by similarity in ecosystems and their type, quality, and quantity of natural resources. Usage of ecoregions aids in structuring and facilitating ecosystem management plans by federal, state, and non-governmental agencies that are responsible for managing the state's environmental resources and services (Woods et al. 2004). The minimum and maximum latitude and longitude values for the state of Arkansas are 32.2°N to 36.5°N for latitude and 89.6°W to 94.6°W for longitude.

### Coordinates

32.2°N and 36.5°N Latitude; 94.6°W and 89.6°W Longitude.

## Taxonomic coverage

### Description

**Figure 3.**
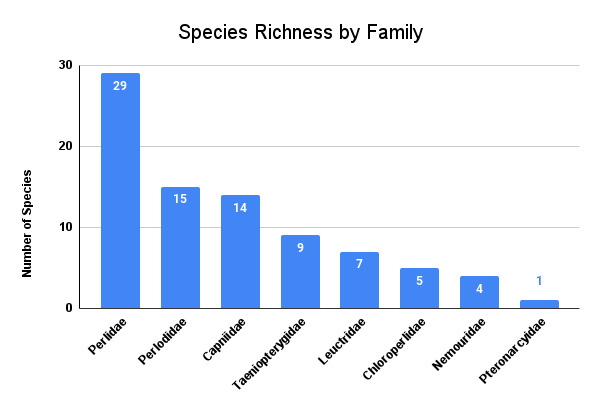
Arkansas stonefly species richness by family.

Herein, we report 84 species of stoneflies, collected from 1,219 unique localities and representing eight families and 24 genera (Table 2). Perlidae contributed 29 of these species, followed distantly by the other families (Fig. 3). In order to visualize historic versus recent collection records, Table 2 provides the number of unique localities for each stonefly species in pre-1980 and post-1980 time frames. We used the year 1980 to distinguish two temporal periods of stonefly collecting efforts because Poulton's extensive collecting was conducted beginning in the 1980s (Fig. 1) (Poulton and Stewart 1991). There are currently nine stonefly species listed as SGCN for the state of Arkansas (Table 2). If we use the total number of unique collection localities to categorize species as rare, we demonstrate that 25 additional species have five or fewer unique collection localities; several might potentially be added to the Arkansas SGCN list. Additional work is needed to determine if any of these species should be added in the future.

**Figure 4.**
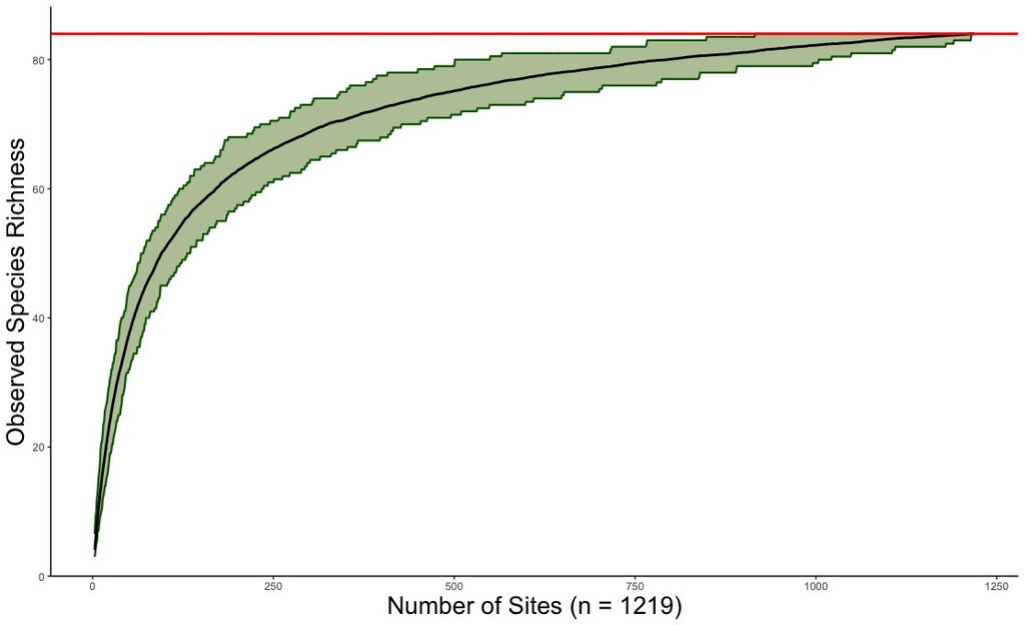
Plecoptera species accumulation curve for the state of Arkansas. Current observed species richness of 84 species is indicated by the red line. The Chao 1 estimate for species richness is illustrated with a solid black line. A 95% confidence interval (range of possible number of species) is indicated by light green shading.

Our assessment of species accumulation suggests that our sampling is nearly complete given the shape of the curve and that Chao1 richness is predicted at 88.0±3.7 species (Fig. 4). Few additional species are likely to be collected with more effort, though several species not included in the list are known from adjacent states.

## Temporal coverage

**Data range:** 1869-1-01 – 2024-5-26.

### Notes

This dataset spans nearly two centuries of specimen collection records. Few stonefly collections occurred before the 1960s (n = 189, ~ 5%). The majority of specimen records were collected during the 1980s - 1990s (n = 2,074, ~ 58%) (Fig. 1). Only a few studies of stonefly distributions of political boundaries within the United States have addressed historical collections (Favret and DeWalt 2002, DeWalt et al. 2005). Those that have addressed historical collections have identified losses in Perlid stoneflies (DeWalt et al. 2005) without limited recolonization. These results are unable to be tested as of yet with the current Arkansas dataset due to the paucity of records post 2000. New efforts are needed to determine if those species reported by Poulton and Stewart (1991) are declining.

## Usage licence

### Usage licence

Open Data Commons Attribution License

## Data resources

### Data package title

The stoneflies (Plecoptera) of Arkansas: a checklist compiled from museum specimen data

### Resource link

https://www.gbif.org/dataset/9040d60a-2486-4f5e-a508-07401ce98f15

### Number of data sets

1

### Data set 1.

#### Data set name

Arkansas Plecoptera V1

#### Data format

Darwin Core Archive

#### Character set

UTF-8

#### Download URL

https://www.gbif.org/dataset/9040d60a-2486-4f5e-a508-07401ce98f15

#### Description

The following dataset presents all of the specimen records included in this preliminary study of the stoneflies of Arkansas. This dataset combines specimen data from 13 collections and several literature sources. The data include mainly identifications to the species rank. Georeferences are provided for most specimens. This data file contains 3,561 records, structured in 77 columns in Darwin Core Archive (DwCA) format. Please use the citation generated by GBIF to cite this dataset.

**Data set 1.** 

Column label	Column description
datasetName	The name identifying the dataset from which the record was derived.
basisOfRecord	The specific nature of the data record.
occurrenceID	A UUID for the dwc:Occurrence (as opposed to a particular digital record of the dwc:Occurrence).
catalogNumber	An identifier (preferably unique) for the record within the dataset or collection.
individualCount	The number of individuals present at the time of the dwc:Occurrence.
preparations	A list (concatenated and separated) of preparations and preservation methods for a dwc:MaterialEntity.
lifeStage	The age class or life stage of the dwc:Organism(s) at the time the dwc:Occurrence was recorded.
sex	The sex of the biological individual(s) represented in the dwc:Occurrence.
country	The name of the country or major administrative unit in which the dcterms:Location occurs.
countryCode	The standard code for the country in which the dcterms:Location occurs.
stateProvince	The name of the next smaller administrative region than country (state, province, canton, department, region, etc.) in which the dcterms:Location occurs.
county	The full, unabbreviated name of the next smaller administrative region than stateProvince (county, shire, department, etc.) in which the dcterms:Location occurs.
eventDate	The date-time or interval during which a dwc:Event occurred. For occurrences, this is the date-time when the dwc:Event was recorded. Not suitable for a time in a geological context.
fieldNumber	An identifier given to the event in the field. Often serves as a link between field notes and the dwc:Event.
minimumElevationInMeters	The lower limit of the range of elevation (altitude, usually above sea level), in meters.
samplingProtocol	The names of, references to, or descriptions of the methods or protocols used during a dwc:Event.
habitat	A category or description of the habitat in which the dwc:Event occurred.
verbatimElevation	The original description of the elevation (altitude, usually above sea level) of the Location.
verbatimEventDate	The verbatim original representation of the date and time information for a dwc:Event.
verbatimLocality	The original textual description of the place.
verbatimLabel	The content of this term should include no embellishments, prefixes, headers or other additions made to the text. Abbreviations must not be expanded and supposed misspellings must not be corrected. Lines or breakpoints between blocks of text that could be verified by seeing the original labels or images of them may be used. Examples of material entities include preserved specimens, fossil specimens, and material samples. Best practice is to use UTF-8 for all characters. Best practice is to add comment “verbatimLabel derived from human transcription” in dwc:occurrenceRemarks.
waterBody	The name of the water body in which the dcterms:Location occurs.
TW:DataAttribute:CollectionObject:waterBody	Custom data attribute field from TaxonWorks (database) in DwCA- The name of the water body in which the dcterms:Location occurs.
publicLands	Custom field- PNHPC field indicating the name of the park or public lands in which the locality occurs.
recordedBy	A person, group, or organization responsible for recording the original dwc:Occurrence.
recordedByID	A list (concatenated and separated) of the globally unique identifier for the person, people, groups, or organizations responsible for recording the original dwc:Occurrence.
identifiedBy	A list (concatenated and separated) of names of people, groups, or organizations who assigned the dwc:Taxon to the subject.
identifiedByID	A list (concatenated and separated) of the globally unique identifier for the person, people, groups, or organizations responsible for assigning the dwc:Taxon to the subject.
dateIdentified	The date on which the subject was determined as representing the dwc:Taxon.
nomenclaturalCode	The nomenclatural code (or codes in the case of an ambiregnal name) under which the dwc:scientificName is constructed.
kingdom	The full scientific name of the kingdom in which the dwc:Taxon is classified.
phylum	The full scientific name of the phylum or division in which the dwc:Taxon is classified.
class	The full scientific name of the class in which the dwc:Taxon is classified.
order	The full scientific name of the order in which the dwc:Taxon is classified.
higherClassification	A list (concatenated and separated) of taxa names terminating at the rank immediately superior to the referenced dwc:Taxon.
superfamily	The full scientific name of the superfamily in which the dwc:Taxon is classified.
family	The full scientific name of the family in which the dwc:Taxon is classified.
subfamily	The full scientific name of the subfamily in which the dwc:Taxon is classified.
genus	The full scientific name of the genus in which the dwc:Taxon is classified.
specificEpithet	A brief phrase or a standard term ("cf.", "aff.") to express the determiner's doubts about the dwc:Identification.
scientificName	The full scientific name, with authorship and date information if known. When forming part of a dwc:Identification, this should be the name in lowest level taxonomic rank that can be determined. This term should not contain identification qualifications, which should instead be supplied in the dwc:identificationQualifier term.
scientificNameAuthorship	The authorship information for the dwc:scientificName formatted according to the conventions of the applicable dwc:nomenclaturalCode.
taxonRank	The taxonomic rank of the most specific name in the dwc:scientificName.
identificationQualifier	A brief phrase or a standard term ("cf.", "aff.") to express the determiner's doubts about the dwc:Identification.
typeStatus	A list (concatenated and separated) of nomenclatural types (type status, typified scientific name, publication) applied to the subject.
institutionCode	The name (or acronym) in use by the institution having custody of the object(s) or information referred to in the record.
institutionID	An identifier for the institution having custody of the object(s) or information referred to in the record.
verbatimCoordinates	The verbatim original spatial coordinates of the dcterms:Location. The coordinate ellipsoid, geodeticDatum, or full Spatial Reference System (SRS) for these coordinates should be stored in dwc:verbatimSRS and the coordinate system should be stored in dwc:verbatimCoordinateSystem.
verbatimLatitude	The verbatim original latitude of the dcterms:Location. The coordinate ellipsoid, geodeticDatum, or full Spatial Reference System (SRS) for these coordinates should be stored in dwc:verbatimSRS and the coordinate system should be stored in dwc:verbatimCoordinateSystem.
verbatimLongitude	The verbatim original longitude of the dcterms:Location. The coordinate ellipsoid, geodeticDatum, or full Spatial Reference System (SRS) for these coordinates should be stored in dwc:verbatimSRS and the coordinate system should be stored in dwc:verbatimCoordinateSystem.
decimalLatitude	The geographic latitude (in decimal degrees, using the spatial reference system given in dwc:geodeticDatum) of the geographic center of a dcterms:Location. Positive values are north of the Equator, negative values are south of it. Legal values lie between -90 and 90, inclusive.
decimalLongitude	The geographic longitude (in decimal degrees, using the spatial reference system given in dwc:geodeticDatum) of the geographic center of a dcterms:Location. Positive values are east of the Greenwich Meridian, negative values are west of it. Legal values lie between -180 and 180, inclusive.
footprintWKT	A Well-Known Text (WKT) representation of the shape (footprint, geometry) that defines the dcterms:Location. A dcterms:Location may have both a point-radius representation (see dwc:decimalLatitude) and a footprint representation, and they may differ from each other.
coordinateUncertaintyInMeters	The horizontal distance (in meters) from the given dwc:decimalLatitude and dwc:decimalLongitude describing the smallest circle containing the whole of the dcterms:Location. Leave the value empty if the uncertainty is unknown, cannot be estimated, or is not applicable (because there are no coordinates). Zero is not a valid value for this term.
georeferenceProtocol	A description or reference to the methods used to determine the spatial footprint, coordinates, and uncertainties.
georeferenceRemarks	Notes or comments about the spatial description determination, explaining assumptions made in addition or opposition to those formalised in the method referred to in dwc:georeferenceProtocol.
georeferenceSources	A map, gazetteer, or other resource used to georeference the dcterms:Location.
georeferencedBy	A list (concatenated and separated) of names of people, groups, or organizations who determined the georeference (spatial representation) for the dcterms:Location.
georeferencedDate	The date on which the dcterms:Location was georeferenced.
occurrenceStatus	A statement about the presence or absence of a dwc:Taxon at a dcterms:Location.
associatedMedia	A list (concatenated and separated) of identifiers (publication, global unique identifier, URI) of media associated with the dwc:Occurrence.
occurrenceRemarks	Comments or notes about the dwc:Occurrence.
eventRemarks	Comments or notes about the dwc:Event.
CollectingEventConcatenate	Custom field- Collecting event data string created by using concatenate.
reference	Custom field- The literature reference in which the record or specimen is cited.
referencePage	Custom field- The page on which the literature references the record or specimen.
Region	Custom field- The biogeographic realm in which the specimen was collected.
vernacularName	A common or vernacular name.
geodeticDatum	The ellipsoid, geodetic datum, or spatial reference system (SRS) upon which the geographic coordinates given in dwc:decimalLatitude and dwc:decimalLongitude are based.
verbatimSRS	The ellipsoid, geodetic datum, or spatial reference system (SRS) upon which coordinates given in dwc:verbatimLatitude and dwc:verbatimLongitude, or dwc:verbatimCoordinates are based.
accessRights	A Dublin Core term denoting information about who can access the resource or an indication of its security status.
lvh:CNC:Group	Custom field- CNC field for group.
lvh:CNC:modifiedBy	Custom field- CNC field for person who last modified the specimen record.
lvh:CNC:modifiedOn	Custom field- CNC field for the date of which the specimen record was modified.
lvh:CNC:createdBy	Custom field- CNC field for person who created the specimen record.
lvh:CNC:createdOn	Custom field- CNC field for the date of which the specimen record was created.
beginDateReared	Custom field- PNHPC field for the date of which the specimen had started to be reared.

## Supplementary Material

A637C726-5893-586C-9950-BBB1C925595B 10.3897/BDJ.13.e145637.suppl1 Supplementary material 1 Arkansas Plecoptera V1 Data type occurrences Brief description The following dataset presents all specimen records included in this preliminary study of the stoneflies of Arkansas. It combines specimen record data from Illinois Natural History Survey Insect Collection, Canadian National Collection of Insects, Western Kentucky University, and several other institutional, personal, and literature records. The data include specimens to their lowest possible taxonomic rank based on identification and determination. The data include geographic coordinates for all specimens where label data permitted--some locations were georeferenced to the mouth of the stream or to a county centroid; those with less information were not georeferenced.
This data file is available for download from Global Biodiversity Information Facility (GBIF). Please import the tsv file into your preferred spreadsheet program. These data are composed of 3,561 records, structured in 77 columns of data in DwCA format. Many specimens used in this work were previously digitized and geo-referenced for a project that was supported by the United States National Science Foundation: CSBR: Natural History: Securing Alcohol Types and Donated Alcohol Specimens at the INHS Insect Collection NSF DBI: CSBR 14-58285. File: oo_1210750.tsv https://binary.pensoft.net/file/1210750 Lily V. Hart, R. Edward DeWalt, Phillip N. Hogan, Scott A. Grubbs, David K. Burton

## Figures and Tables

**Table 1.** Dataset summary. Summary of origin of specimen records in Arkansas Plecoptera V1, the number of species found in each, range of years of collections, and record count for each source.

**Record Origin (institutionCode)**	**Institution Name**	**Species Represented**	**Temporal Range**	**Record Count**
INHS	Illinois Natural History Survey Insect Collection	80	1869—2022	3124
CNCI	Canadian National Collection of Insects, Arachnids and Nematodes	27	1937—1972	95
BYU	Brigham Young University Life Sciences Museum	9	1962—1999	27
CM	Carnegie Museum of Natural History	1	1985	1
CSUIC	C.P. Gillette Museum of Arthropod Diversity	8	1978—2003	19
WKU	Western Kentucky University	6	1990—2020	20
ISIC	Iowa State University Insect Collection	2	1965	6
MSU	A.J. Cook Arthropod Research Collection, Michigan State University	2	1958-—2001	7
ND	Museum of Biodiversity, University of Notre Dame	4	1975—1977	5
OSUM	C.A. Triplehorn Insect Collection, Ohio State University	2	1964—1968	2
PERC	Purdue University Entomological Research Collection	2	1960—1974	2
WIRC	Wisconsin Insect Research Collection, University of Wisconsin-Madison	1	1937	1
PNHPC_data	Phillip N. Hogan Personal Collection	21	2022—2024	160
Literature_data		24	1958—2002	92
**TOTAL**				**3,561**

**Table 2.** Checklist of Arkansas stoneflies and the number of unique localities for pre-1980 and post-1980, and in total. Bold text indicates those species listed as Species of Greatest Conservation Need (AWAP 2015). Species marked with an asterisk (*) are known from only five or fewer unique localities. Species marked with an obelisk (†) reflect taxonomic and nomenclatural updates. Please see text for more information.

**Family**	**Species**	**Unique localities pre-1980**	**Unique localities post-1980**	**Total**
Capniidae	*Allocapniagranulata* (Claassen, 1924)	29	54	83
Capniidae	***Allocapniajeanae* Ross, 1964**	6	13	19
Capniidae	***Allocapniamalverna* Ross, 1964**	1	29	30
Capniidae	*Allocapniamohri* Ross & Ricker, 1964	50	109	159
Capniidae	*Allocapniamystica* Frison, 1929	8	34	42
Capniidae	***Allocapniaoribata* Poulton & Stewart, 1987**	1	8	9
Capniidae	***Allocapniaozarkana* Ross, 1964**	6	3	9
Capniidae	*Allocapniapeltoides* Ross & Ricker, 1964*	1	4	5
Capniidae	*Allocapniarickeri* Frison, 1942	36	116	152
Capniidae	*Allocapniasandersoni* Ricker, 1952	10	6	16
Capniidae	*Allocapniavivipara* (Claassen, 1924)*	0	1	1
Capniidae	***Allocapniawarreni* Ross & Yamamoto, 1966**	1	0	1
Capniidae	*Nemocapniacarolina* Banks, 1938*	1	0	1
Capniidae	*Paracapniaangulata* Hanson, 1961	2	4	6
Chloroperlidae	***Alloperlacaddo* Poulton & Stewart, 1987**	0	4	4
Chloroperlidae	*Alloperlacaudata* Frison, 1934	5	4	9
Chloroperlidae	*Alloperlahamata* Surdick, 1981*	0	2	2
Chloroperlidae	*Alloperlaouachita* Stark & Stewart, 1983*	0	4	4
Chloroperlidae	*Haploperlabrevis* (Banks, 1895)	13	16	29
Leuctridae	***Leuctraszczytkoi* Stark & Stewart, 1981**†	0	10	10
Leuctridae	*Leuctratenuis* (Pictet, 1841)	2	20	22
Leuctridae	*Zealeuctracherokee* Stark & Stewart, 1973	0	9	9
Leuctridae	*Zealeuctraclaasseni* (Frison, 1929)	20	26	46
Leuctridae	*Zealeuctranarfi* Ricker & Ross, 1969	3	19	22
Leuctridae	***Zealeuctrawachita* Ricker & Ross, 1969**	2	3	5
Leuctridae	*Zealeuctrawarreni* Ricker & Ross, 1969	16	24	40
Nemouridae	*Amphinemuradelosa* (Ricker, 1952)	7	14	21
Nemouridae	*Amphinemuratexana* Baumann, 1996*†	0	2	2
Nemouridae	*Prostoiaozarkensis* Baumann & Grubbs, 2014†	17	18	35
Nemouridae	*Shipsarotunda* (Claassen, 1923)*	0	2	2
Perlidae	*Acroneuriaevoluta* Klapálek, 1909†	4	8	12
Perlidae	*Acroneuriafilicis* Frison, 1942	2	5	7
Perlidae	*Acroneuriafrisoni* Stark & Brown, 1991†	7	23	30
Perlidae	*Acroneuriainternata* (Walker, 1852)*	0	1	1
Perlidae	*Acroneuriaozarkensis* Poulton & Stewart, 1991*	1	1	2
Perlidae	*Acroneuriaperplexa* Frison, 1937	6	12	18
Perlidae	*Agnetinacapitata* (Pictet, 1841)*	2	1	3
Perlidae	*Agnetinaflavescens* (Walsh, 1862)	5	7	12
Perlidae	*Attaneuriaruralis* (Hagen, 1861)*	2	0	2
Perlidae	*Neoperlacarlsoni* Stark & Baumann, 1978	0	6	6
Perlidae	*Neoperlacatharae* Stark & Baumann, 1978	3	3	6
Perlidae	*Neoperlachoctaw* Stark & Baumann, 1978	0	9	9
Perlidae	*Neoperlafalayah* Stark & Lentz, 1988	7	9	16
Perlidae	*Neoperlaharpi* Ernst & Stewart, 1986	3	15	18
Perlidae	*Neoperlaosage* Stark & Lentz, 1988	8	15	23
Perlidae	*Neoperlarobisoni* Poulton & Stewart, 1986	4	7	11
Perlidae	*Paragnetina kansensis* (Banks, 1905)*	0	2	2
Perlidae	*Paragnetina media* (Walker, 1852)*	0	2	2
Perlidae	*Perlestabaumanni* Stark, 1989*	1	4	5
Perlidae	*Perlestabolukta* Stark, 1989	2	13	15
Perlidae	*Perlestabrowni* Stark, 1989	1	12	13
Perlidae	*Perlestacinctipes* (Banks, 1905)	1	5	6
Perlidae	*Perlestadecipiens* (Walsh, 1862)	2	20	22
Perlidae	*Perlestaephelida* Grubbs & DeWalt, 2012*	0	3	3
Perlidae	*Perlestafusca* Poulton & Stewart, 1991*	0	2	2
Perlidae	*Perlestalagoi* Stark, 1989	4	6	10
Perlidae	*Perlestasublobata* South & DeWalt, 2019	5	21	26
Perlidae	*Perlinelladrymo* (Newman, 1839)	3	5	8
Perlidae	*Perlinellaephyre* (Newman, 1839)	4	6	10
Perlodidae	*Clioperlaclio* (Newman, 1839)	4	21	25
Perlodidae	*Helopicusnalatus* (Frison, 1942)	0	6	6
Perlodidae	*Hydroperlacrosbyi* (Needham & Claassen, 1925)	4	9	13
Perlodidae	*Hydroperlafugitans* (Needham & Claassen, 1925)*	1	0	1
Perlodidae	*Isoperlabilineata* (Say, 1823)*	2	0	2
Perlodidae	*Isoperlaburksi* Frison, 1942*	0	5	5
Perlodidae	*Isoperladavisi* James, 1974	0	6	6
Perlodidae	*Isoperladecepta* Frison, 1935*	0	1	1
Perlodidae	*Isoperladicala* Frison, 1942*	0	2	2
Perlodidae	*Isoperlairregularis* (Klapálek, 1923)	3	18	21
Perlodidae	*Isoperlanamata* Frison, 1942	11	19	30
Perlodidae	*Isoperlaouachita* Stark & Stewart, 1973	13	26	39
Perlodidae	*Isoperlarichardsoni* Frison, 1935*	0	1	1
Perlodidae	*Isoperlasignata* (Banks, 1902)*	0	1	1
Perlodidae	***Isoperlaszczytkoi* Poulton & Stewart, 1987**	0	1	1
Pteronarcyidae	*Pteronarcyspictetii* Hagen, 1873*	0	4	4
Taeniopterygidae	*Strophopteryxarkansae* Ricker & Ross, 1975	5	16	21
Taeniopterygidae	*Strophopteryxcucullata* Frison, 1934	3	20	23
Taeniopterygidae	*Strophopteryxfasciata* (Burmeister, 1839)	20	59	79
Taeniopterygidae	*Taeniopteryxburksi* Ricker & Ross, 1968	33	43	76
Taeniopterygidae	*Taeniopteryxlita* Frison, 1942	5	22	27
Taeniopterygidae	*Taeniopteryxlonicera* Ricker & Ross, 1968*	1	1	2
Taeniopterygidae	*Taeniopteryxmaura* (Pictet, 1841)	9	17	26
Taeniopterygidae	*Taeniopteryxmetequi* Ricker & Ross, 1968	13	28	41
Taeniopterygidae	*Taeniopteryxparvula* Banks, 1918	8	22	30

## References

[B12190046] Annaratone B., Larson C., Prater C., Dowling A., Magoulick D. D., Evans-White M. A. (2023). Predicting habitat and distribution of an Interior Highlands regional endemic Winter Stonefly (*Allocapniamohri*) in Arkansas using Random Forest Models. Hydrobiology.

[B12033455] Baumann R. W. (1996). Three new species of *Amphinemura* (Plecoptera: Nemouridae) from eastern North America. Entomological News.

[B12556515] Delpeuch Antonin, Morris Tom, Huynh David, (bot) Weblate, Mazzocchi Stefano, Jacky, Guidry Thad, elebitzero, Stephens Owen, Matsunami Isao, Sproat Iain, Larsson Albin, Santos Silvério, Mayer Allana, kushthedude, Fauconnier Sandra, Mishra Ekta, Magdinier Martin, Beaubien Antoine, Liu Lu, Tacchelli Fabio, Ong Joanne, Giroud Florian, Nordhøy Allan, [Sannita] Luca Martinelli, Kanye Elroy, Saby Mathieu, Chandra Lisa (2024). OpenRefine/OpenRefine: OpenRefine 3.8.7. Zenodo.

[B12065286] DeWalt R. E., Favret C., Webb D. W. (2005). Just how imperiled are aquatic insects? A case study of stoneflies (Plecoptera) in Illinois. Annals of the Entomological Society of America.

[B11945025] DeWalt R. E., Yoder M., Snyder E., Dmitriev D., Ower Geoffrey (2018). Wet collections accession: a workflow based on a large stonefly (Insecta, Plecoptera) donation. Biodiversity Data Journal.

[B11947256] DeWalt R. E., Hopkins H., Neu-Becker U., Stueber G. Plecoptera Species File. https://plecoptera.speciesfile.org.

[B12560368] ESRI (2024). ArcGIS Pro version 3.3.1.

[B12065277] Favret C., DeWalt R. E. (2002). Comparing the Ephemeroptera and Plecoptera specimen databases at the Illinois Natural History Survey and using them to document changes in the Illinois fauna. Annals of the Entomological Society of America.

[B11993465] Fowler A., Anderson J. (2015). The Arkansas Wildlife Action Plan. https://www.agfc.com/wp-content/uploads/2023/04/Arkansas-Wildlife-Action-Plan.pdf.

[B12098355] Frison T. H. (1934). Four new species of stoneflies from North America (Plecoptera). The Canadian Entomologist.

[B12004610] Frison T. H. (1942). Studies of North American Plecoptera. Illinois Natural History Survey Bulletin.

[B12097863] Grubbs S. A., DeWalt R. E. (2012). *Perlestaephelida*, a new Nearctic stonefly species (Plecoptera, Perlidae). ZooKeys.

[B12095373] Grubbs S. A., Kondratieff B. C., Stark B. P., DeWalt R. E. (2013). A review of the Nearctic genus *Zealeuctra* Ricker (Plecoptera, Leuctridae), with the description of a new species from the Cumberland Plateau region of eastern North America. ZooKeys.

[B12095364] Grubbs S. A., Baumann R. W., DeWalt R. E., Tweddale T. (2014). A review of the Nearctic genus *Prostoia* (Ricker) (Plecoptera, Nemouridae), with the description of a new species and a surprising range extension for *P.hallasi* Kondratieff & Kirchner. ZooKeys.

[B11993474] Grubbs S. A., DeWalt R. Edward, Hart L. V., Layer M. R. (2023). Systematics and updated range alter the conservation status of the Louisiana Needlefly, *Leuctraszczytkoi* Stark & Stewart, 1981 (Plecoptera: Leuctridae). Zoosymposia.

[B12244631] Guevara L., Vargas-Cuenca J., Hortelano-Moncada Y., Cervantes F. A. (2024). A specimen-based database of small-eared shrews (Mammalia, Eulipotyphla, Cryptotis) in the Neotropical Region. Biodiversity Data Journal.

[B12190057] Harp George L., Robison Henry W. (2006). Aquatic macroinvertebrates of the Strawberry River system in North-Central Arkansas. Journal of the Arkansas Academy of Science.

[B12203236] Hogan Phillip N., DeWalt R. Edward, Grubbs Scott A. (2024). The female and emended male description of the USA Interior Highlands endemic *Allocapniaoribata* Poulton & Stewart, 1987 (Plecoptera: Capniidae). Journal of Insect Biodiversity.

[B12558356] Illies J. (1966). Katalog der rezenten Plecoptera. Das Tierreich – Eine Zusammenstellung und Kennzeichnung der rezenten Tierformen.

[B12558366] Needham J. G., Claassen P. W. (1925). A Monograph of the Plecoptera or Stoneflies of America North of Mexico. The Thomas Say Foundation.

[B12269723] Newman Evan A., DeWalt R. Edward, Grubbs Scott A. (2021). Plecoptera (Insecta) Diversity in Indiana: A Watershed-Based Analysis. Diversity.

[B12017661] Oksanen J., Simpson G., Blanchet F., Kindt R., Legendre P., Minchin P., O'Hara R., Solymos P., Stevens M., Szoecs E., Wagner H., Barbour M., Bedward M., Bolker B., Borcard D., Carvalho G., Chirico M., De Caceres M., Durand S., Evangelista H., FitzJohn R., Friendly M., Furneaux B., Hannigan G., Hill M., Lahti L., McGlinn D., Ouellette M., Ribeiro Cunha E., Smith T., Stier A., Ter Braak C., Weedon J. (2024). _vegan: Community Ecology Package_. R package version 2.6-6.1. https://CRAN.R-project.org/package=vegan.

[B12269745] Team Posit (2024). RStudio: Integrated Development Environment for R. Posit Software. http://www.posit.co/.

[B11945035] Poulton B. P., Stewart K. W. (1987). Three new species of stoneflies (Plecoptera) from the Ozark-Ouachita Mountain region. Proceedings of the Entomological Society of Washington.

[B11945044] Poulton B. P., Stewart K. W. (1991). The stoneflies of the Ozark and Ouachita Mountains (Plecoptera). Memoirs of the American Entomological Society.

[B12050723] Ricker W. E. (1952). Systematic studies in Plecoptera. Indiana University Publications Science Series.

[B12049915] Ricker W. E., Ross H. H. (1968). North American species of *Taeniopteryx* (Plecoptera, Insecta). Journal of the Fisheries Research Board of Canada.

[B12049924] Ricker W. E., Ross H. H. (1969). The genus *Zealeuctra* and its position in the family Leuctridae (Plecoptera, Insecta). Canadian Journal of Zoology.

[B12560155] Ricker W. E., Ross H. H. (1975). Synopsis of the Brachypterinae, (Insecta: Plecoptera: Taeniopterygidae). *Canadian Journal of Zoology*.

[B11947245] Robison H. W., McAllister C. T. (2018). A preliminary checklist of the stoneflies (Arthropoda: Insecta: Plecoptera) of Arkansas. Journal of the Arkansas Academy of Science.

[B12017651] Ross H. H. (1964). New species of Winter Stoneflies of the genus *Allocapnia* (Plecoptera, Capniidae). Entomological News.

[B11945053] Ross H. H., Ricker W. E. (1971). The classification, evolution, and dispersal of the winter stonefly genus *Allocapnia*. Illinois biological monographs.

[B12045361] South E. J., DeWalt R. E., Davis M. A., Thomas M. J. (2019). A new stonefly species (Plecoptera, Perlidae) from the Interior Highlands USA, with morphological and molecular comparison to other congeneric species. ZooKeys.

[B12010923] Stark Bill P., Brown Lynda D. (1991). What is *Acroneuriaevoluta* Klapálek (Plecoptera: Perlidae)?. Aquatic Insects.

[B11945062] Stark B. P. (2004). Perlidae (The Stones). In Stark & Armitage. The stoneflies (Plecoptera) of eastern North America. Volume II. Chloroperlidae, Perlidae, Perlodidae (Perlodinae).. Ohio Biological Survey Bulletin New Series.

[B12065214] Szczytko S. W., Kondratieff B. C. (2015). A review of the Eastern Nearctic Isoperlinae (Plecoptera: Perlodidae) with the description of twenty-two species. Monographs of Illiesia.

[B12033497] Community TaxonWorks (2022). TaxonWorks [software and supporting resources], https://taxonworks.org.. https://github.com/SpeciesFileGroup/taxonworks..

[B12033374] Survey United States Geological Highest and Lowest Elevations. https://www.usgs.gov/educational-resources/highest-and-lowest-elevations.

[B12278305] USEPA Level III Ecoregions of Arkansas.. https://gaftp.epa.gov/epadatacommons/ORD/Ecoregions/ar/ar_eco_l3.htm.

[B12017698] Wickham H. (2016). ggplot2: Elegant Graphics for Data Analysis.

[B12556404] Wieczorek J., Bloom D., Guralnick R., Blum S., Döring M. (2012). Darwin Core: An Evolving Community-Developed Biodiversity Data Standard. PLoS ONE.

[B12033276] Woods A. J., Foti T. L., Chapman S. S., Omernik J. M., Wise J. A., Murray E. O., Prior W. L., Pagan J. B., Comstock Jr. J. A., Radford M. (2004). Ecoregions of Arkansas (color poster with map, descriptive text, summary tables, and photographs).

